# Unraveling the Rural Lived Experience With Dementia: Pathways to Improved Care.

**DOI:** 10.1093/geroni/igaf122.1597

**Published:** 2025-12-31

**Authors:** Joshua Fergen, Dana Ketcher, Melinda Dertinger, Kelsie Larson, Kirsten Cruikshank, Amy Otto, Wayne Warry, Kristen Jacklin

**Affiliations:** University of Minnesota Medical School, Duluth Campus, Duluth, Minnesota, United States; University of Minnesota Medical School, Duluth Campus, Duluth, Minnesota, United States; University of Minnesota Medical School, Duluth Campus, Duluth, Minnesota, United States; University of Minnesota Medical School Memory Keepers Medical Discovery Team, Duluth, Minnesota, United States; University of Minnesota Medical School Memory Keepers Medical Discovery Team, Duluth, Minnesota, United States; University of Minnesota Medical School Memory Keepers Medical Discovery Team, Duluth, Minnesota, United States; University of Minnesota Medical School, Duluth Campus, Duluth, Minnesota, United States; University of Minnesota Medical School, Duluth Campus, Duluth, Minnesota, United States

## Abstract

This study describes findings from a qualitative, community-based participatory research project with rural communities. Using ethnographic techniques and a phenomenological approach, our aim was to collect and analyze data focused on the lived experience of dementia in rural communities to inform appropriate program development and interventions. Community-based researchers conducted 63 interviews with formal dementia care providers and community-dwelling older adults in northern rural Minnesota. Data were organized and analyzed using QSR NVivo. Findings identify the socio-cultural and environmental aspects of rural spaces, along with rural Alzheimer’s disease and related dementias (ADRD) care accessibility, as cross-cutting themes to guide a socio-ecological model of care. Results provide insight into needs, barriers and opportunities at the level of the home, clinic and community, that require policy intervention across those three levels. For example, technological solutions in the home, dementia education in the clinic, and addressing spatial isolation in the community. Centering community voices and perspectives revealed gaps in our understanding how rural contexts shape community experiences with ADRD, which can inform actionable strategies and interventions. Results from this study identify the need for educational interventions and clinical approaches to improve care, unique challenges and adaptations that could facilitate aging in place, and assets in rural communities that can support persons living with dementia and their caregivers. These results can be used to create highly tailored programming, interventions, and policies for rural populations that are impacted by ADRD.

